# Lipid emulsion treatment for local anesthetic systemic toxicity in pediatric patients: A systematic review

**DOI:** 10.1097/MD.0000000000037534

**Published:** 2024-03-15

**Authors:** Soo Hee Lee, Sunmin Kim, Ju-Tae Sohn

**Affiliations:** a Department of Anesthesiology and Pain Medicine, Gyeongsang National University Changwon Hospital 11, Gyeongsangnam-do, Republic of Korea; b Department of Anesthesiology and Pain Medicine, Gyeongsang National University College of Medicine, Gyeongsangnam-do, Republic of Korea; c Institute of Medical Science, Gyeongsang National University, Jinju-si, Republic of Korea; d Department of Anesthesiology and Pain Medicine, Gyeongsang National University Hospital, Gyeongsangnam-do, Republic of Korea; e Department of Anesthesiology and Pain Medicine, Gyeongsang National University College of Medicine, Gyeongsang National University Hospital, Gyeongsangnam-do, Republic of Korea.

**Keywords:** arrhythmia, bupivacaine, lipid emulsion, local anesthetic systemic toxicity

## Abstract

**Background:**

Local anesthetic systemic toxicity (LAST) is rare, but fatal; the current widely used treatment is lipid emulsion (LE). The goal of this study was to analyze and review case reports on LE treatment for LAST in pediatric patients.

**Methods:**

We performed a systematic review using case reports on LE treatment for LAST in pediatric patients, searching PubMed and Scopus databases to March 2023 using the following keywords: (“local anesthetic toxicity” OR “local anesthetic systemic toxicity” OR LAST”) AND (“newborn” OR “infant” OR “child” OR “children” OR “adolescent” OR “pediatric”) AND (“lipid emulsion” OR “Intralipid”).

**Results:**

Our search yielded 21 cases, revealing that nearly 43% patients with LAST were less than 1 year old, and most cases were caused by bupivacaine (approximately 67% cases). “Inadvertent intravascular injection” by anesthesiologists and “overdose of local anesthetics” mainly by surgeons were responsible for 52% and 24% cases of LAST, respectively. LAST occurred in the awake state (52%) and under general anesthesia (48%), mainly causing seizures and arrhythmia, respectively. Approximately 55% of patients received LE treatment in <10 minutes after LAST, mainly improving cardiovascular symptoms. A 20% LE (1.5 mL/kg) dose followed by 0.25 mL/kg/minutes dose was frequently used. LE and anticonvulsants were mainly used in the awake state, whereas LE with or without vasopressors was mainly used under general anesthesia. LE treatment led to full recovery from LAST in 20 cases; however, 1 patient died due to underlying disease.

**Conclusion:**

Consequently, our findings reveal that LE is effective in treating pediatric LAST.

## 1. Introduction

Local anesthetics are widely used for peripheral nerve block, local infiltration, and spinal and epidural anesthesia. While the occurrence of local anesthetic systemic toxicity (LAST) is rare, it is fatal. Lipid emulsion, originally developed for parenteral nutrition in 1961, is now widely used to treat LAST.^[1]^ In addition, lipid emulsion was reported to be effective in treating intractable systemic toxicity in pediatric patients caused by non-local anesthetic drugs with a high lipid solubility, which is unresponsive to supportive treatments.^[2]^ The incidence of LAST in children was estimated to be 8 per 100,000 nerve blocks (95% confidence interval: 0.3–1.6). LAST is induced mainly by inadvertent intravascular injection or overdose of local anesthetic; it causes cardiac arrhythmia, myocardial depression, and cardiac arrest via inhibition of cardiac sodium, potassium, and calcium channels.^[3]^ In addition, LAST causes central nervous systemic symptoms such as seizures.^[3]^ Predominantly, regional anesthesia in children is administered while they are under general anesthesia, and diagnosis of LAST may be delayed, increasing the risk of LAST.^[4,5]^ When comparing pharmacokinetics with adults, the pediatric population generally exhibits decreased drug absorption, protein binding of the drug, metabolism, and excretion, whereas volume of distribution of the drug is increased.^[6]^ Amino-amide local anesthetics are metabolized by the hepatic cytochrome P450 enzymes and extensively bind to α1-acid glycoprotein.^[7]^ Neonates and infants have low levels of α1-acid glycoproteins and the immature form of hepatic microsomal enzymes, leading to increased free form of amino-amide local anesthetics and decreased metabolism, respectively.^[5]^ Thus, the risk of LAST in neonates and infants may be increased.^[5]^ Moreover, pediatric patients who were born prematurely, have low muscle mass, and suffer from hepatic or cardiac diseases, may have an increased risk of LAST compared with the healthy pediatric population. Presently, no systematic reviews have been conducted on lipid emulsion treatment for LAST in pediatric patients. Thus, the goal of this study was to analyze and review case reports, retrieved through PubMed and Scopus databases, on lipid emulsion treatment for LAST in pediatric patients (<19 years old).

## 2. Methods

**Figure 1.**
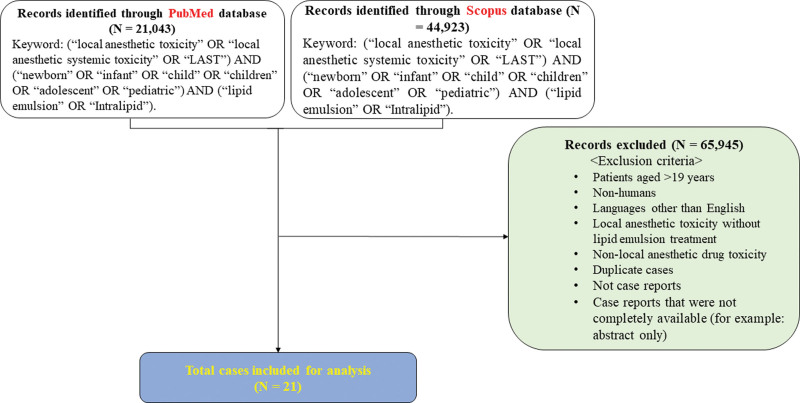
Flow chart demonstrating the search process for case reports on lipid emulsion treatment for local anesthetic systemic toxicity in pediatric patients using the PubMed and Scopus databases. N indicates the number of articles.

A PubMed and Scopus database search was performed for relevant case reports from their respective dates of inception until March 31, 2023, using the following keywords: (“local anesthetic toxicity” OR “local anesthetic systemic toxicity” OR “LAST”) AND (“newborn” OR “infant” OR “child” OR “children” OR “adolescent” OR “pediatric”) AND (“lipid emulsion” OR “Intralipid”). Institutional review board approval was not required as this involved an analysis and review of case reports. We retrieved 21,043 and 44,923 case reports through the PubMed and Scopus databases, respectively (Fig. 1). After applying the exclusion criteria, a total of 21 cases were selected for analysis (Fig. 1). The following data were extracted from each case report: age, body weight, route of administration of the local anesthetic, administration of local anesthetic under general anesthesia, dosage of local anesthetics, presumed cause of LAST, use of ultrasonography, whether anesthesia was induced by anesthesiologists or non-anesthesiologists, symptoms of LAST, administration method of lipid emulsion, lipid emulsion dosage, improvement in symptoms by lipid emulsion, other treatments, and outcomes (Table 1).

**Table 1:** Lipid emulsion treatment for local anesthetic systemic toxicity in pediatric patients.

No	Age	BW (kg)	Route of Adm of LA	Under GA	Dosages of LA	Presumed cause of LAST	Usage of US	Induced by AN or SG	Sx of LAST	Adm method of LE	LE dosage	Improved Sx by LE	Other Tx
1^[8]^	3 mo	5.9	Local Inf	Y	BPV 20 mg LDC 80 mg	Overdose		SG	Seizure, VF, broad-complex arrhythmia	Bolus: 1.5 mL/kg Con: 0.25 mL/kg/min	Bolus: 9 mL, Con: N/A	CV, CNS	Supportive care, ICU care, anticonvulsant,
2^[9]^	6 d	4	ESPB	Y	LBP 1 mg/kg	IIVI	Y	AN	Decreased ETCO_2_	Bolus: 1.5 mL/kg	6 mL	CV	Epi (1 mcg/kg)
3^[10]^	3 mo	3.9	Caudal + IV	Y	BPV 7.5 mg LDC 120 mg	Overdose		AN	Bradycardia, hypotension, seizure	Bolus: 1^st^ 3 mL/kg 2^nd^ 2.56 mL/kg Con: 1 mL/min	144mL	CV, CNS	Intubation, anticonvulsant, ICU care
4^[11]^	11 mo	8	Penile B	Y	BPV 40 mg	Overdose		SG	VT	Bolus: 1.5 mL/kg Con: 0.25 mL/kg/min	Bolus: 12 mL, Con: N/A	CV	Supportive care
5^[12]^	17 yr	N/A	Axillary B	N	BPV 75 mg	IIVI		AN	Seizure, VA	Bolus: 1^st^ 100 mL 2^nd^ 100 mL Con: N/A	Bolus 200 mL Con: N/A	CV	Anticonvulsant, CPR, ICU care
6^[13]^	11 yr	55.6	MNB	N	MPV 54 mg	IIVI		SG	Light headedness, seizure, numbness, emesis	Bolus: 1^st^ 1 mL/kg 2^nd^ 1 mL/kg 3^rd^ 1 mL/kg Con: 0.25 mL/kg/min	Bolus: 166.8 mL Con: 5838 mL	CNS	Anticonvulsant
7^[14]^	17 mo	10	Caudal	Y	LBP 2.5 mg/kg	IIVI		AN	ST depression, T-wave elevation, Hypotension	Bolus: 1 mL/kg	10 mL	CV	Supportive care
8^[15]^	4 mo	6.54	Dermal	N	EMLA (LDC 1500 mg, and PLC 1500 mg)	Overdose		SG	Seizure, respiratory depression, hypoxia, methemoglobinemia	Bolus: 5 mL/kg	32.7 mL	CNS	Supportive care, methylene blue
9^[16]^	2 mo	4.8	Caudal	N	BPV 2.12 5mg/kg	IIVI		AN	Seizure, apnea, SVT, BP dropped	Bolus: 1.25 mL/kg Con: 0.26 mL/kg/min	Bolus 6 mL Con: 75 mL/hr	CNS, CV	Supportive care, anticonvulsant
10^[17]^	2 d	3.2	Caudal	Y	BPV 8 mg	IIVI	Y	AN	Multifocal PVC, bradycardia	Bolus: 1 mL/kg	3.2 mL	CV	CPR
11^[18]^	13 yr	55	PLPB	Y	LDC 10 0 mg RPV75 mg	IIVI		AN	Tachycardia, VA, wide QRS	Bolus: 3 mL/kg	165 mL	CV	None
12^[19]^	17 yr	61	FNB	N	BPV 100 mg	IIVI		AN	Seizure, pulseless activity of ECG, VA	Bolus: N/A, Con: N/A	500 mL (8 mL/kg)	CNS, CV	Anticonvulsant, supportive care, CPR, defibrillation
13^[20]^	2 mo	12	ECB	N	BPV 22.5 mg LDC 120 mg	Overdose		SG	Seizure, desaturation, tachycardia, T-wave & ST elevation	Bolus: 6 mL	6 mL	CNS, CV	Supportive care, anticonvulsant,
14^[21]^	40 d	4.96	Caudal	Y	BPV 8.75 mg	IIVI		AN	Tachycardia, ST depression, decreased ETCO_2,_ hypotension, hypoxia	Bolus: 2 mL/kg	9.92 mL	CV	Epi, albumin, ICU care
15^[22]^	3 yr	11	Caudal	Y	BPV 25 mg	IIVI		AN	Pulseless VA	Bolus: 1^st^ 1.36 mL/kg 2^nd^ 0.45 mL/kg Con: 0.23 mL/kg/min	Bolus: 20 mL Con: N/A	CV	Supportive care, CPR, Epi
16^[23]^	18 yr	86	Epidural	N	BPV 65 mg	IIVI		AN	Restless, agitated, unresponsive, twitching, tachycardia,	Bolus: 1^st^ 0.58 mL/kg 2^nd^ 0.58 mL/kg 3^rd^ 4.65 mL/kg		CNS	Anticonvulsant
17^[24]^	6 yr	24	Epidural	N	BPV 12 mg	Secondary carnitine deficiency		AN	Bradycardia, wide complex VA, sinus tachycardia	Bolus: 0.8 mL/kg Con: 0.25 mL/kg/min	Bolus 20 mL Con: 192 mL	CV	CPR, vasopressor, ICU care
18^[25]^	15 mo	N/A	Oral	N	EMLA (LDC, and PLC), N/A	Ingestion			Seizure, hypoxia	Bolus: 1.5 mL/kg Con: 0.25 mL/kg/min	N/A	CNS	Anticonvulsant, NaHCO_3_, methylene blue
19^[19]^	17 yr	61	FNB	N	BPV 100 mg	IIVI		AN	N/A	N/A	N/A	N/A	Defibrillation, CPR, anticonvulsant
20^[26]^	16 yr	58	ISB	N	LDC 30 mg RPV 300 mg	Overdose	Y	AN	Confusion, visual hallucinations, slightly slurred speech, non-coordinated tremor, increased RR, slightly elevated BP	Bolus: 1.5 mL/kg Con: 0.25 mL/kg/min	Bolus 87 mL Con: 2610 mL	CNS, CV	None
21^[27]^	6 yr	N/A	Local Inf	Y	BPV 7.5 mg/kg	Overdose		SG	Bradycardia, asystole	Bolus: 1.5 mL/kg Con: 0.25 mL/kg/min	N/A	CV	Vasopressor

Adm = Administration, AN = Anesthesiologist, AXB = axillary block, B = block, BP = blood pressure, BPV = bupivacaine, BW = body weight, CNS = central nervous system, Con = continuous infusion, CPR = cardiopulmonary resuscitation, CV = cardiovascular system, d = days, D = death, ECB = extraconeal block, Epi = Epinephrine, ESPB = erector spinae plane block, ETCO_2_ = end-tidal carbon dioxide tension, FNB = femoral nerve block, FR = full recovery, GA = general anesthesia, hr = hour, ICU = intensive care unit, IIVI = inadvertently intravascular injection, Inf = Infiltration, ISB = interscalene block, LA = local anesthetics, LAST = local anesthetic systemic toxicity, LBP = levobupivacaine, LDC = lidocaine, LE = lipid emulsion, MNB = mandibular nerve block, mo = month, N/A = not available, N = none, No = number, PLC = prilocaine, PLPB = posterior lumbar plexus block, PVC = premature ventricular contraction, RPV = ropivacaine, RR = respiratory rate, SG = surgeon, SVT = supraventricular tachycardia, Sx = symptom, Tx = treatment, Under GA = administration of local anesthetics under general anesthesia, US = Ultrasound, VA = ventricular arrhythmia, VF = ventricular fibrillation, VT = ventricular tachycardia, Y = yes, yr = years.

## 3. Results

A total of 21 case reports (i.e., data pertaining to 21 patients) on lipid emulsion treatment for LAST in pediatric patients were retrieved through PubMed and Scopus databases (Table 1).

### 3.1. Age and sex distribution

**Figure 2.**
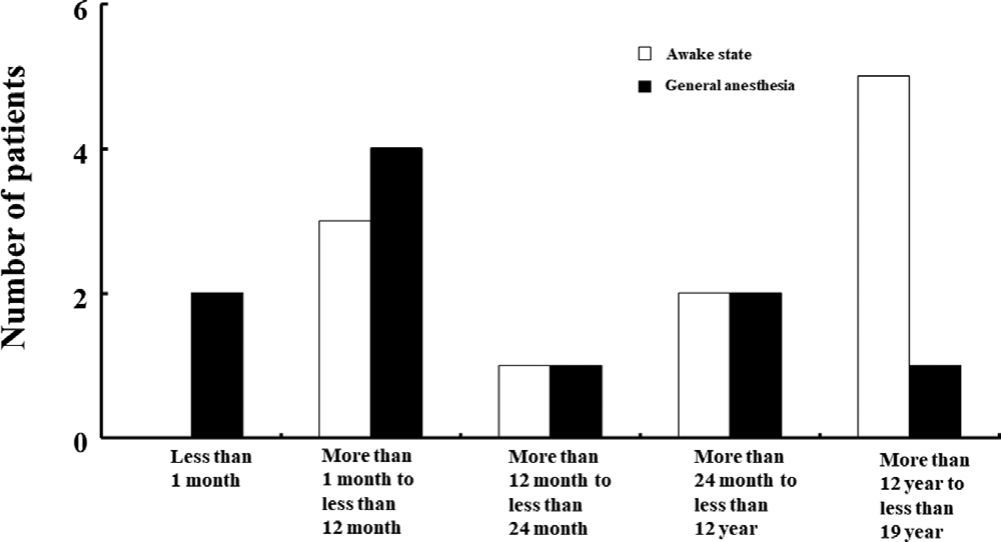
Age distribution of pediatric patients (N = 21) that underwent lipid emulsion treatment for local anesthetic systemic toxicity in the awake state (N = 11) and under general anesthesia (N = 10). N indicates the number of patients.

Age distribution of patients is shown in Figure 2. The most common age group was < 1 year (<1 month of age, n = 2 patients; ≥1 month and < 12 months of age, n = 7) (Fig. 2). Eleven male and 7 female patients received lipid emulsion treatment for LAST. Information regarding sex was not available for 3 patients.

### 3.2. Different local anesthetics associated with LAST

**Figure 3.**
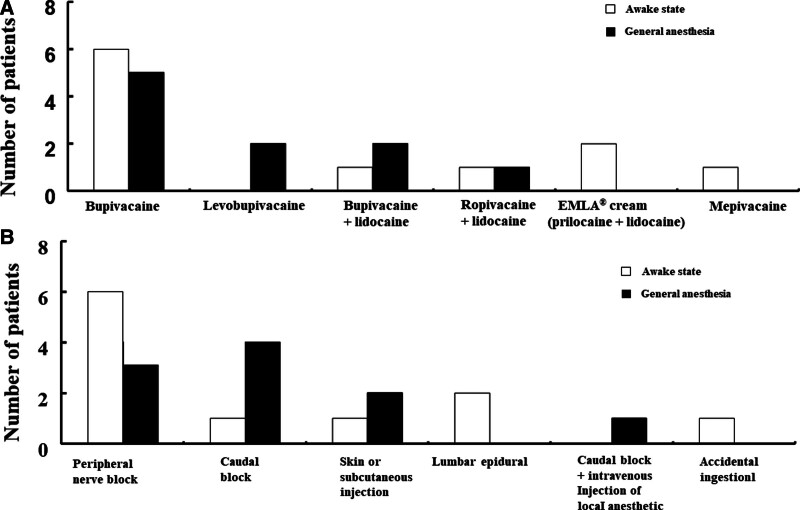
(A) Distribution of local anesthetics causing local anesthetic systemic toxicity (LAST) in pediatric patients (N = 21) that underwent lipid emulsion treatment for LAST in the awake state (N = 11) and under general anesthesia (N = 10). N indicates the number of patients. (B) The route of administration of local anesthetics in pediatric patients (N = 21) that underwent lipid emulsion treatment for LAST in the awake state (N = 11) and under general anesthesia (N = 10). N indicates the number of patients.

The most common anesthetic associated with pediatric LAST was bupivacaine (n = 14; either bupivacaine administered alone [n = 11] or bupivacaine plus lidocaine administered in combination [n = 3]) (Fig. 3A). In pediatric patients, other local anesthetics that caused LAST were levobupivacaine (n = 2), ropivacaine plus lidocaine (n = 2), EMLA cream (prilocaine plus lidocaine, n = 2), and mepivacaine (n = 1) (Fig. 3A).

### 3.3. Route of administration of local anesthetics and measures to prevent LAST

The routes of local anesthetic administration commonly associated with LAST in pediatric patients were peripheral nerve block (n = 9) and caudal block (n = 5) (Fig. 3B). Peripheral nerve block (Table 1) encompassed erector spinae block (n = 1), femoral nerve block (n = 2), axillary nerve block (n = 1), penile block (n = 1), mandibular nerve block (n = 1), lumbar plexus block (n = 1), extracorneal block (n = 1), and interscalene block (n = 1). Of the 9 peripheral nerve blocks, 6 were performed by anesthesiologists and 3 (mandibular nerve block, extracorneal block, and penile block) by non-anesthesiologists. Local anesthesia was administered under ultrasound guidance in only 3 patients by anesthesiologists (Table 1). In 17 patients, regional anesthesia was performed without ultrasound guidance or information regarding usage of ultrasound was not available. The measures that were undertaken to prevent LAST other than administration of regional anesthesia under ultrasound guidance were as follows (Table 1): negative aspiration test alone (n = 8), negative aspiration and epinephrine (n = 3), and epinephrine alone (n = 1). In the remaining 8 patients, other measures were not performed or information regarding other measures was not available. As 1 patient accidentally ingested EMLA cream (Case No. 18), this patient was excluded from the analysis of measures to prevent LAST.

### 3.4. LAST induced by anesthesiologists versus non-anesthesiologists and presumed causes of LAST

The administration of local anesthesia by anesthesiologists and non-anesthesiologists (surgeons or dentists) led to 14 and 7 cases of LAST in pediatric patients, respectively. Herein, patients that developed LAST after receiving less than the maximum recommended dose of local anesthetic were considered to have received an “inadvertent intravascular injection of local anesthetics” and those that received more than the recommended dose were considered to have “overdose of local anesthetics.” Occurrence of LAST in 1 patient (Case No. 17) appeared to be owing to secondary carnitine deficiency induced by valproic acid treatment. In addition, the cause of LAST in another patient (Case No. 18) was due to accidental ingestion of EMLA cream. Thus, these 2 cases were excluded from the analysis of presumed causes of LAST. Following were found to be the presumed causes of LAST: inadvertent intravascular injection of local anesthetics (n = 12; by anesthesiologists [n = 11] and by a dentist, i.e., non-anesthesiologist [n = 1]) and overdose of local anesthetics (n = 7; by anesthesiologists [n = 2] and by surgeons, i.e., non-anesthesiologists [n = 5]). The detailed information regarding overdose of local anesthetics is as follows: 20 mg bupivacaine and 80 mg lidocaine were used for local infiltration in a patient weighting 5.9 kg (Case No. 1); 120 mg intravenous lidocaine was used for the treatment of laryngospasm in a patient weighting 3.9 kg (Case No. 3); 40 mg bupivacaine was used for penile block in a patient weighting 8 kg (Case No. 4); EMLA cream (1500 mg lidocaine and prilocaine) was used for dermal application in a patient weighting 6.54 kg (Case No. 8); 22.5 mg bupivacaine and 120 mg lidocaine were used for extracorneal block in a patient weighting 12 kg (Case No. 13); 30 mg lidocaine and 300 mg ropivacaine were used for interscalene block in a patient weighting 58 kg (Case No. 20); and 7.5 mg/kg bupivacaine was used for donor site infiltration in a 6-year-old burn patient (Case No. 21). In pediatric patients, LAST occurred in the operation room, including the recovery room, and in the non-operating room in 17 and 4 cases, respectively.

### 3.5. Lipid emulsion treatment

**Figure 4.**
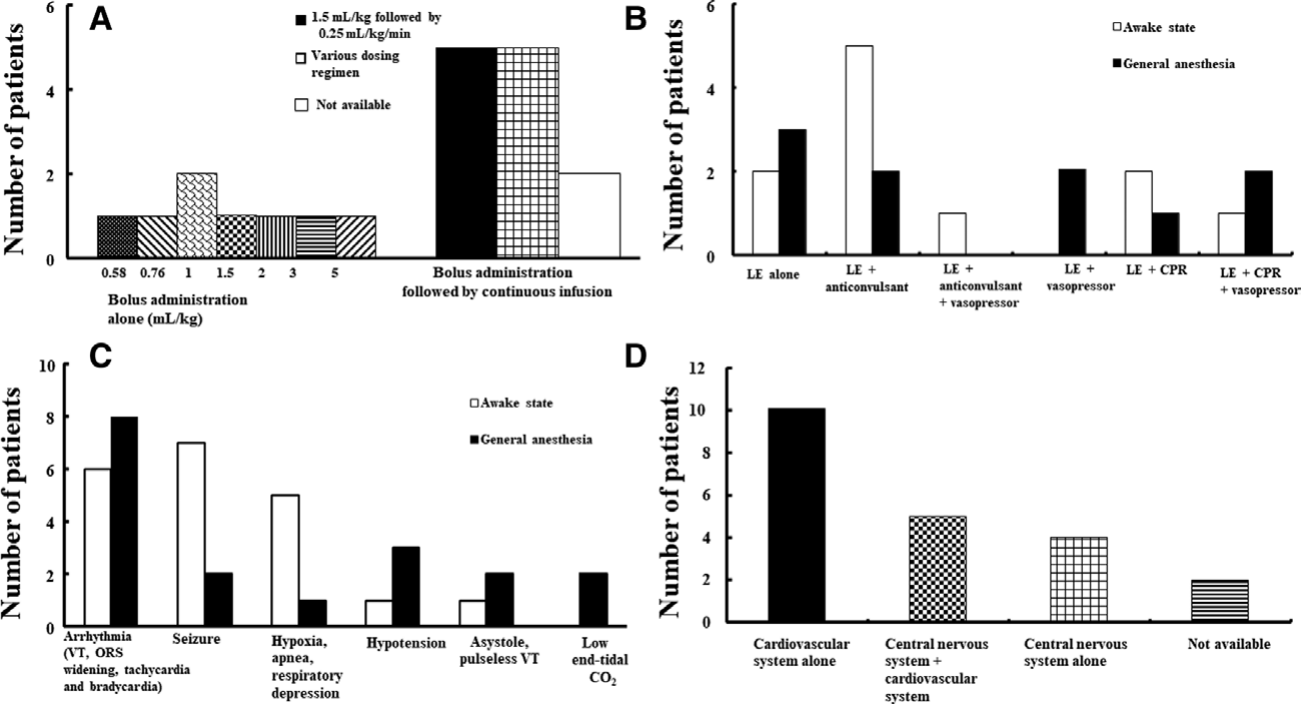
(A) Methods of lipid emulsion (LE) administration for pediatric local anesthetic systemic toxicity (LAST, N = 20). (B) Treatment patterns for LAST using LE with or without other treatments in pediatric LAST (N = 21). (C) Occurrence of various symptoms due to LAST in pediatric patients that underwent LE treatment for LAST in the awake state and under general anesthesia. (D) Improvement in symptoms (N = 21) post LE treatment. N indicates the number of patients. CO2 = carbon dioxide tension, CPR = cardiopulmonary resuscitation, VT = ventricular tachycardia.

Eight patients received only bolus administration of lipid emulsion, and 12 patients received bolus administration of lipid emulsion followed by continuous infusion (Fig. 4A). However, information regarding lipid emulsion administration was not available for 1 patient. Regarding bolus administration of 20% lipid emulsion alone, doses of 0.58, 0.76, 1, 1.5, 2, 3, and 5 mL/kg were used in 1, 1, 2, 1, 1, 1, and 1 patient, respectively (Fig. 4A). Furthermore, 42% of patients, who underwent bolus administration followed by continuous infusion, received 20% lipid emulsion (1.5 mL/kg) followed by 0.25 mL/kg/minutes of continuous infusion (Fig. 4A). Following supportive treatments were generally used for pediatric LAST: airway maintenance (oxygen supply, intubation, and ventilator care), fluid administration, and sodium bicarbonate administration. Lipid emulsion treatment was used with or without other treatments in patients undergoing supportive treatments (Fig. 4B). Lipid emulsion and anticonvulsants (n = 5) were mainly used in the awake state (Fig. 4B). Lipid emulsion alone (n = 3) or lipid emulsion plus vasopressor was mainly used under general anesthesia (Fig. 4B). Duration from LAST to administration of lipid emulsion was as follows: lipid emulsion treatment < 5 minutes after LAST (n = 8) and lipid emulsion treatment from ≥ 5 to < 10 minutes after LAST (n = 3). Furthermore, 2 patients each received lipid emulsion from “≥10 to < 30 min,” “≥30 min to ≤ 1 h,” and “>1 h” after LAST. Information regarding duration for lipid emulsion administration after LAST was not available for 4 patients. Duration from lipid emulsion administration to improved symptoms were as follows: immediately (n = 6), <4 minutes (n = 4), ≥4 minutes to < 1 hour (n = 4), and ≥ 1 hour (n = 2). Information regarding duration for improved symptoms after lipid emulsion administration was not available for 5 patients. The following symptoms were mainly observed in pediatric patients with LAST (Fig. 4C): arrhythmia including ventricular tachycardia, QRS widening, tachycardia and bradycardia (n = 14), seizures (n = 9), and hypoxia, apnea, and respiratory depression (n = 6) (Fig. 4C). Symptom improvements after lipid emulsion treatment for pediatric LAST were as follows (Fig. 4D): improvement in cardiovascular symptoms alone (n = 10); improvement in both cardiovascular and central nervous system symptoms (n = 5); and improvement in central nervous symptoms alone (n = 4). Information regarding symptom improvement was not available for 2 patients. After lipid emulsion treatment, 20 pediatric patients fully recovered from LAST, but 1 patient died (Table 1). Intralipid was used in 15 patients with LAST and Lipovenous in 1 patient. Five case reports just described it as “lipid emulsion.”

### 3.6. Side effects of lipid emulsion treatment

Only 1 patient (Case No. 6) showed lipemic blood and elevated serum triglyceride levels (16,583 mg/dL) due to a large amount of injected lipid emulsion (66 mL/kg).

### 3.7. LAST and its treatment under general anesthesia

The 10 cases of LAST occurred under general anesthesia in the operating room (Figs. 2 and 3); of these, 6 patients were infants (<1 year old). The presumed causes of LAST under general anesthesia were inadvertent intravascular injection of local anesthetics (by anesthesiologist [n = 6]) and overdose of local anesthetics (n = 4, by anesthesiologist [n = 1] and by surgeon [n = 3]) (Table 1). The presumed symptoms of LAST under general anesthesia were as follows: cardiovascular symptoms (n = 10); low end-tidal carbon dioxide tension (n = 2); central nervous symptoms (seizures, n = 2); and hypoxia (n = 1). Peripheral nerve block and other procedures performed under general anesthesia, which are associated with LAST, were as follows: caudal block (n = 5); local infiltration of anesthetics in the wound site (n = 2), elector spinae plane block (n = 1), penile block (n = 1), and posterior lumbar plexus block (n = 1). Statistics of lipid emulsion treatment with or without other treatments are shown in Figure 4B.

### 3.8. Methemoglobinemia

LAST induced by EMLA cream (1500 mg lidocaine and 1500 mg prilocaine) in a 4-month-old patient caused methemoglobinemia (methemoglobin = 22.8%), which was treated with methylene blue (1.5 mg/kg) (Case No. 8).

## 4. Discussion

This systematic review was conducted to analyze and review lipid emulsion treatment for LAST in pediatric patients; our findings indicate that lipid emulsion is effective in treating LAST in pediatric patients. The main findings of the present investigation can be summarized as follows: bupivacaine (used in approximately 67% of included cases) was commonly associated with LAST; LAST in patients for whom anesthesia was administered by anesthesiologists was mainly due to inadvertent intravascular injection of local anesthetics, whereas LAST in patients for whom anesthesia was administered by non-anesthesiologists was mainly due to overdose of local anesthetics; symptoms of LAST frequently included arrhythmia under general anesthesia and seizures in the awake state; lipid emulsion mainly improved cardiovascular symptoms (in approximately 71% of cases); and lipid emulsion and anticonvulsants were frequently used to treat LAST in the awake state, and lipid emulsion with or without vasopressors was frequently used to treat LAST in patients treated under general anesthesia.

Local anesthetics cause myocardial depression in the following order: bupivacaine > ropivacaine > lidocaine.^[28]^ In addition, the low ratio of “local anesthetic dose required to produce cardiovascular collapse” to “local anesthetic dose required to produce central nervous system toxicity” is associated with high cardiotoxicity, whereas a high ratio is associated with relatively low cardiotoxicity.^[29]^ This ratio increases in anesthetics in the following order: bupivacaine, levobupivacaine, and ropivacaine.^[29]^ In children, bupivacaine has been reported to be the most common anesthetic causing LAST.^[4]^ Similar to this finding, the present review revealed that bupivacaine most commonly caused LAST, which was treated with lipid emulsion.^[4]^ Thus, alternative local anesthetics such as levobupivacaine and ropivacaine should be considered to prevent bupivacaine-induced LAST. Inadvertent intravascular injection of local anesthetics was mostly associated with anesthesia administered by anesthesiologists (in approximately 92% of cases involving inadvertent intravascular injection of local anesthetics), whereas overdose of local anesthetics was mostly associated with anesthesia administered by non-anesthesiologists (in approximately 71% of cases involving overdose of local anesthetics). Vascularity of the injection site of the local anesthetic is known to be associated with LAST.^[3,30]^ Thus, caudal block, which accounted for approximately 29% of cases of LAST in the present review, may be associated with the high vascularity of the injection site of local anesthetics. Ultrasound-guided nerve block reduces the incidence of LAST and neurologic symptoms.^[31]^ However, in present review, to prevent LAST in pediatric patients, only 4 cases used ultrasound with regional anesthesia administered by anesthesiologists. In addition, the negative aspiration technique with local anesthetics with or without epinephrine, an alternative measure to ultrasound to prevent LAST, was mainly used (in approximately 52% of cases) in the present review. Intravascular injection of epinephrine-containing lidocaine has been reported to produce increased heart rate and T-wave amplitude in children anesthetized with servoflurane.^[32]^ However, in agreement with a previous report, use of negative needle aspiration with or without epinephrine did not always prevent LAST in the present review.^[4]^ According to the previous report, negative aspiration is associated with considerable inaccuracies, and the reaction to adrenaline might lack clinical significance in pediatric cases and other perioperative stimulatory contexts.^[4]^ The following measures are recommended to prevent LAST: evaluation of risk factors such as low body mass (e.g., in infants) and underlying diseases; performing ultrasound-guided nerve blocks; gradual administration of local anesthetic in small increments; needle or catheter aspiration prior to administering local anesthesia; using epinephrine (0.5 μg/kg) in children to detect intravascular injection; and adhering to the maximal recommended doses of local anesthetics.^[33]^ The maximal recommended doses (mg/kg) of local anesthetics without epinephrine are as follows: bupivacaine, 2 mg/kg; levobupivacaine, 2 mg/kg; ropivacaine, 3 mg/kg; mepivacaine, 4.5 mg/kg; lidocaine, 4.5 mg/kg; and prilocaine 6 mg/kg.^[34]^ In addition, the maximum recommended dose of lidocaine and prilocaine is 300 and 400 mg, respectively.^[30]^ Taking into consideration this maximum recommended dose, the doses of bupivacaine that led to LAST in the present review were found to be approximately 1.69, 2.5, and 3.75 times the maximum recommended dose of bupivacaine. The doses of lidocaine that led to LAST were approximately 2.22 and 3.01 times the maximum recommended dose of lidocaine. The doses of lidocaine and prilocaine contained in EMLA cream that led to LAST in the present review were 5 and 3.75 times their maximum recommended doses, respectively. The dose of ropivacaine that led to LAST was approximately 1.73 times the maximum recommended dose. Therefore, the results of the present review suggest that non-anesthesiologists, specially, should adhere to the maximum recommended doses of local anesthetics to prevent LAST.

LAST frequently occurs in infants (<1-year-olds).^[4,35]^ Similar to previous reports, this review indicates that children < 1 year of age (approximately 43% of included cases) frequently experienced LAST and underwent lipid emulsion treatment.^[4,35]^ Approximately 67 percent of pediatric patients < 1 year of age in the present review experienced LAST under general anesthesia. In addition, α1-acid glycoprotein level increases with age until 6 months.^[36]^ In the current review, the putative risk factors associated with the high incidence of LAST in pediatric patients < 1 year of age may be reduced α1-acid glycoprotein and immature hepatic cytochrome P450 enzyme levels.^[5,36]^

LAST produces central nervous symptoms, such as perioral numbness, slurred speech, and tinnitus, followed by convulsion, coma, and respiratory depression.^[3]^ In addition, it produces cardiovascular symptoms, such as hypertension and tachycardia followed by hypotension, myocardial depression, bradycardia, ventricular tachycardia, and cardia arrest.^[3]^ A previous report on LAST in children stated that initial presentation of LAST in the awake state and under general anesthesia mainly involves central nervous system and cardiovascular system symptoms, respectively.^[4]^ Similarly, in the present review, the most frequently occurring LAST symptom under general anesthesia was arrhythmia and in the awake state was seizures.^[4]^ The previous study reported that 68% of instances of LAST in children occurred under anesthesia.^[4]^ Similarly, in present review, 48% of LAST cases occurred under general anesthesia.^[4]^ Additionally. consistent with the previous report, all patients who developed LAST under general anesthesia experienced cardiovascular symptoms.^[4]^ Decreased end-tidal carbon dioxide tension were observed as one of the signs of LAST in 2 cases under general anesthesia, which may be attributed to the decreased venous return caused by local anesthetic-induced myocardial depression. Cardiovascular symptoms showed most improvement (approximately 55% of included cases) after lipid emulsion treatment for non-local anesthetic toxicity in pediatric patients.^[2]^ Similarly, after lipid emulsion treatment, the maximum improvement was seen in cardiovascular symptoms alone (48%), followed by symptoms of both central nervous and cardiovascular systems, and central nervous system symptoms alone.^[2]^ Before LAST progresses to cardiovascular collapse, early administration of lipid emulsion produces leads to recovery from central nervous system symptoms induced by ropivacaine toxicity, and shortens recovery time from unconsciousness induced by lidocaine toxicity.^[37,38]^ In addition, lipid emulsion treatment was originally recommended to treat intractable cardiac arrest induced by LAST; however, early administration of lipid emulsion is now recommended to treat the incipient stage of LAST.^[33]^ According to this recommendation, approximately 36% of patients received lipid emulsion treatment <5 minutes after LAST, and approximately 55% of patients received lipid emulsion treatment < 10 minutes after LAST.^[33]^ In addition, approximately 45% of patients showed improvement in symptoms in < 4 minutes after lipid emulsion administration for LAST, and approximately 67% of patients showed improvement in symptoms in < 1 hour after lipid emulsion administration for LAST. Treatment of LAST includes airway maintenance to prevent hypoxia and acidosis, lipid emulsion and benzodiazepines to treat seizures, low doses of epinephrine (1 μg/kg), advanced cardiac life support for cardiac arrest, and cardiopulmonary bypass.^[1]^ According to the present review, lipid emulsion and anticonvulsants were frequently used to treat LAST in the awake state (Fig. 4B), whereas lipid emulsion alone and lipid emulsion plus vasopressor were frequently used to treat LAST under general anesthesia (Fig. 4B).

The underlying mechanism of lipid emulsion treatment for LAST is lipid shuttle, increased myocardial contractility, provision of fatty acid, inhibition of mitochondrial dysfunction, attenuation of nitric oxide release, and glycogen synthase kinase-3β phosphorylation.^[1]^ The most widely accepted mechanism of lipid emulsion treatment for LAST is lipid shuttle; it states that administration of lipid emulsion produces lipid compartments of lipid emulsion, which absorb lipid-soluble local anesthetics (for example: bupivacaine, log P [octanol/water partition coefficient] = 3.41) from the brain and heart, and subsequently, lipid emulsion including the lipid-soluble local anesthetic is carried to the liver, muscle, and adipose tissue, where it undergoes detoxification and is stored.^[1]^ The decreasing order of lipid solubility in local anesthetics is as follows: levobupivacaine (log *P* = 3.6), bupivacaine (log *P* = 3.41), ropivacaine (log *P* = 2.9), lidocaine (log *P* = 2.44), and mepivacaine (log *P* = 1.95).^[1]^ Moreover, lipid emulsion absorbs lipid-soluble local anesthetics and inhibits vasodilation induced by toxic doses of local anesthetics according to lipid solubility.^[39–41]^ In the present review, LAST occurred most frequently with bupivacaine and lipid emulsion treatment was administered. Thus, we surmised that this seems to be due to highest lipid solubility and high cardiotoxicity of bupivacaine.

The recommended dose of lipid emulsion for LAST is as follows: initial bolus administration of 20% lipid emulsion (1.5 mL/kg) over 2 to 3 minutes followed by continuous infusion of 20% lipid emulsion (0.25 mL/kg/min).^[1]^ Consistent with this recommendation, 20% lipid emulsion (1.5 mL/kg) bolus administration followed by 0.25 mL/kg/minutes continuous infusion was frequently used in this review (in approximately 42% of cases involving bolus administration followed by continuous infusion). As propofol is dissolved in 10% lipid emulsion and produces cardiovascular depression, propofol should not be administered instead of 20% lipid emulsion because of the cardiovascular depression exacerbated by propofol itself. The most common type of lipid emulsion used in the treatment for LAST in the present review was Intralipid (in approximately 71% of cases), which is a 100% long-chain fatty acid. However, the availability of Intralipid may be limited in hospitals compared with other types of lipid emulsions, because linoleic acid contained in Intralipid induces proinflammatory mediators.^[42,43]^ Moreover, alternative lipid emulsions such as SMOFlipid, Lipofundin MCT/LCT, and Clinoleic are used to treat LAST.^[44]^ Thus, alternative lipid emulsions other than Intralipid can be used to treat LAST in pediatric patients. Approximately 95% of patients undergoing lipid emulsion treatment in the current review fully recovered from LAST. However, even with lipid emulsion treatment, 1 patient with secondary carnitine deficiency due to valproic acid treatment (Case No. 17) died, and this may be attributed to increased susceptibility to bupivacaine toxicity owing to impaired carnitine shuttle, and subsequently, decreased fatty acid β-oxidation and adenosine triphosphate production in cardiac mitochondria.^[45]^ Lipid emulsion treatment as an antidote produces the following side effects, which interfere with laboratory examination; lipidemia, pancreatitis, adult respiratory distress syndrome, and fat overload.^[46]^ However, these side effects of lipid emulsion treatment are relatively mild compared with LAST. The daily recommended dose of 20% lipid emulsion for parenteral nutrition in pediatric and adult patients is 15 and 12.5 mL/kg, respectively.^[47]^ Consequently, hyperlipidemia and hypertriglyceridemia were observed in only 1 case (Case No. 6) owing to the administration of a high dose (66 mL/kg) of lipid emulsion.

Local anesthetics (benzocaine and lidocaine) and EMLA cream cause methemoglobinemia.^[48,49]^ As methylene blue is converted to leukomethylene blue by nicotinamide adenine dinucleotide phosphate, and leukomethylene blue converts methemoglobin to hemoglobin, methylene blue is used to treat methemoglobinemia.^[48]^ Methemoglobinemia induced by EMLA cream was treated with methylene blue (Case No. 8).

The present review has the following limitations. As a positive case report is relatively easy to be published compared with negative case reports, the bias of positive case reports should be considered while interpreting these results. The number of included case reports in this review was small, limiting generalizability. Thus, further studies with database registries on lipid emulsion treatment for pediatric LAST are needed.

## 5. Conclusion

Lipid emulsion treatment was found to be effective in treating LAST for pediatric patients. Lipid emulsion plus anticonvulsants were predominantly used to treat seizures observed in LAST in the awake state, whereas lipid emulsion alone or lipid emulsion plus vasopressors were predominantly used to treat arrhythmia observed in LAST under general anesthesia. Consequently, lipid emulsion therapy may be a lifesaving intervention for children who experience LAST.

## Author contributions

**Conceptualization:** SooHee Lee, Sunmin Kim, Ju-Tae Sohn.

**Data curation:** SooHee Lee, Sunmin Kim.

**Formal analysis:** SooHee Lee, Sunmin Kim, Ju-Tae Sohn.

**Funding acquisition:** Ju-Tae Sohn.

**Investigation:** SooHee Lee, Sunmin Kim, Ju-Tae Sohn.

**Methodology:** SooHee Lee, Ju-Tae Sohn.

**Project administration:** Ju-Tae Sohn.

**Resources:** SooHee Lee, Sunmin Kim, Ju-Tae Sohn.

**Software:** SooHee Lee, Ju-Tae Sohn.

**Supervision:** Ju-Tae Sohn.

**Visualization:** Ju-Tae Sohn.
